# Giant Hepatic Hemangioma With Kasabach–Merritt Syndrome: Is the Appropriate Treatment Enucleation or Liver Transplantation?

**DOI:** 10.1155/2000/25954

**Published:** 2000-08

**Authors:** Steven N. Hochwald, Leslie H. Blumgart

**Affiliations:** 1 Department of Surgery University of Florida College of Medicine Gainesville Florida USA; 2 Hepatobiliary Service Department of Surgery Memorial Sloan-Kettering Cancer Center 1275 York Avenue New York New York 10021 USA

## Abstract

We present a case of giant cavernous hemangioma of the liver with disseminated intravascular coagulopathy (Kasabach–Merritt syndrome) which was cured by enucleation. The 51 year old woman presented with increased abdominal girth and easy bruisability. Workup elsewhere revealed a massive hepatic hemangioma and she was started on radiation therapy to the lesion and offered an orthotopic liver transplant. After careful preoperative preparation, we felt that resection was possible and she underwent a successful enucleation. The operation and postoperative course were complicated by bleeding but she recovered and remains well in followup after 6 months. All coagulation parameters have returned to normal. Enucleation should be considered the treatment of choice for hepatic hemangiomas, including those presenting with Kasabach–Merritt syndrome. The benefits of enucleation as compared to liver transplantation for these lesions are discussed.


*HPB Surgery*, 2000, Vol. 11, pp. 413–419
Reprints available directly from the publisher
Photocopying permitted by license only

© 2000 OPA (Overseas Publishers Association) N.V.
Published by license under
the Harwood Academic Publishers imprint,
part of The Gordon and Breach Publishing Group.
Printed in Malaysia.

### *Case Report*

### Giant Hepatic Hemangioma with Kasabach–Merritt Syndrome: Is the Appropriate Treatment Enucleation or Liver Transplantation?

STEVEN N. HOCHWALD[a] and LESLIE H. BLUMGART[b,*]

[a]*Department of Surgery, University of Florida College of Medicine, Gainesville, Florida;* [b]*Hepatobiliary Service, Department of Surgery, Memorial Sloan-Kettering Cancer Center, 1275 York Avenue, New York, New York 10021*

*(Received 22 October 1999)*

**We present a case of giant cavernous hemangioma of the liver with disseminated intravascular coagulopathy (Kasabach–Merritt syndrome) which was cured by enucleation. The 51 year old woman presented with increased abdominal girth and easy bruisability. Workup elsewhere revealed a massive hepatic hemangioma and she was started on radiation therapy to the lesion and offered an orthotopic liver transplant. After careful preoperative preparation, we felt that resection was possible and she underwent a successful enucleation. The operation and postoperative course were complicated by bleeding but she recovered and remains well in followup after 6 months. All coagulation parameters have returned to normal. Enucleation should be considered the treatment of choice for hepatic hemangiomas, including those presenting with Kasabach–Merritt syndrome. The benefits of enucleation as compared to liver transplantation for these lesions are discussed.**

*Keywords:* Kasabach-Merritt syndrome, enucleation, hemangioma

### INTRODUCTION

In 1940, Kasabach and Merritt reported a case of thrombocytopenic purpura associated with a rapidly growing cutaneous capillary hemangioma in a 2-month old child [1]. To date more than 100 cases of Kasabach-Merritt syndrome have been described, most in infants with cutaneous hemangiomas. The cardinal features of the Kasabach-Merritt syndrome include an enlarging hemangioma, thrombocytopenia and microangiopathic hemolytic anemia with acute or chronic consumptive coagulopathy. Hepatic cavernous hemangiomas presenting with this syndrome have been infrequently encountered and their management is challenging and controversial. Recently, two cases of orthotopic liver transplant for unresectable hepatic

*Corresponding author. Tel.: (212) 639-5526, Fax: (212) 794-5852.

hemangiomas with Kasabach–Merritt syndrome have been described [2, 3]. We report a case of successful hepatic tumor enucleation for a patient with Kasabach–Merritt syndrome who was considered unresectable and offered liver transplantation before referral.

### Case Report

A 51-year-old female was referred to a hospital for evaluation of increased abdominal girth and fatigue over a 6 month period. In addition, she noticed she had been bruising easily for approximately 2 years. An investigation, including abdominal ultrasound, computed tomographic (CT) scan and liver-spleen scan, revealed a large cavernous hemangioma of the liver. Routine blood tests revealed a prothrombin time of 15–16 seconds (INR 1.3–1.4), a platelet count of 92,000/$mm^3$ and a white blood cell count of 2200/$mm^3$. Careful hematologic evaluation revealed a low fibrinogen level of 69 mg/dL with an elevated D-dimer level of 8.0 mg/dl. The patient's liver function tests were noted to be normal and her albumin level was 4.3 g/dl. Other medical history was significant only for hysterectomy at the age of 28 for heavy bleeding. She had been on estrogen replacement for 10 years.

The patient was evaluated by an outside institution and was felt to be not a suitable candidate for liver resection. She was offered a liver transplant and was started on external beam radiation to the lesion. Further investigations were performed consisting of magnetic resonance imaging (MRI), celiac and superior mesenteric artery angiography, and inferior vena cava and right hepatic venography. These studies revealed an extremely large mass arising from the liver and occupying the entire right side of the abdomen (Fig. 1). Cross sectional imaging revealed the entire left liver to be replaced by this mass. In addition, a separate large mass was present in the caudate lobe (Fig. 2). There was no aberrant arterial supply to the liver. The left portal vein did not fill and there was compression of the main portal vein. There was severe compression of the retrohepatic inferior vena cava (Fig. 3). The left hepatic vein was completely compressed but the right hepatic vein was widely patent (Fig. 4). It was considered that resection was feasible.

After preoperative transfusion of platelets and fresh frozen plasma, laparotomy was performed. A very large hemangioma extended from the cupula of the diaphragm down into the pelvis bilaterally. The large tumor occupied segments I, II, III, IV, V and VIII and was extremely vascular. The left lobe of the liver was first fully mobilized and the right lobe partially mobilized. The hepatic artery was located, noted to be markedly enlarged and cross clamped. The liver was then manually compressed in order to partially empty it of blood, decrease its size and make it more manageable. The right anterior branch of the hepatic artery was ligated. The left hepatic artery was divided between clamps and the left branch of the portal vein was transected. The left hepatic duct was circled with a suture and divided. The hepatic veins could not be reached at this stage of the procedure and it was decided to enucleate the tumor between segments V and VIII and VI and VII. The left and middle hepatic veins were divided as they were reached and segments II, III, IV, V and VIII were removed as an extended left hepatic lobectomy. The caudate lobe, which was markedly enlarged with tumor, was now mobilized from the underlying vena cava and removed almost in its entirety but a small portion of the hemangioma was left just above the right hepatic vein. Final hemostasis was secured with suture ligation and the Argon beam coagulator. Blood loss during this procedure was considerable and amounted to about 10 liters. The patient was transferred to the recovery room in stable condition. Approximately one hour after reaching the recovery room, the patient became hypotensive and was noted to have an expanding abdomen. The patient was taken back to the operating room and exploration revealed multiple areas of

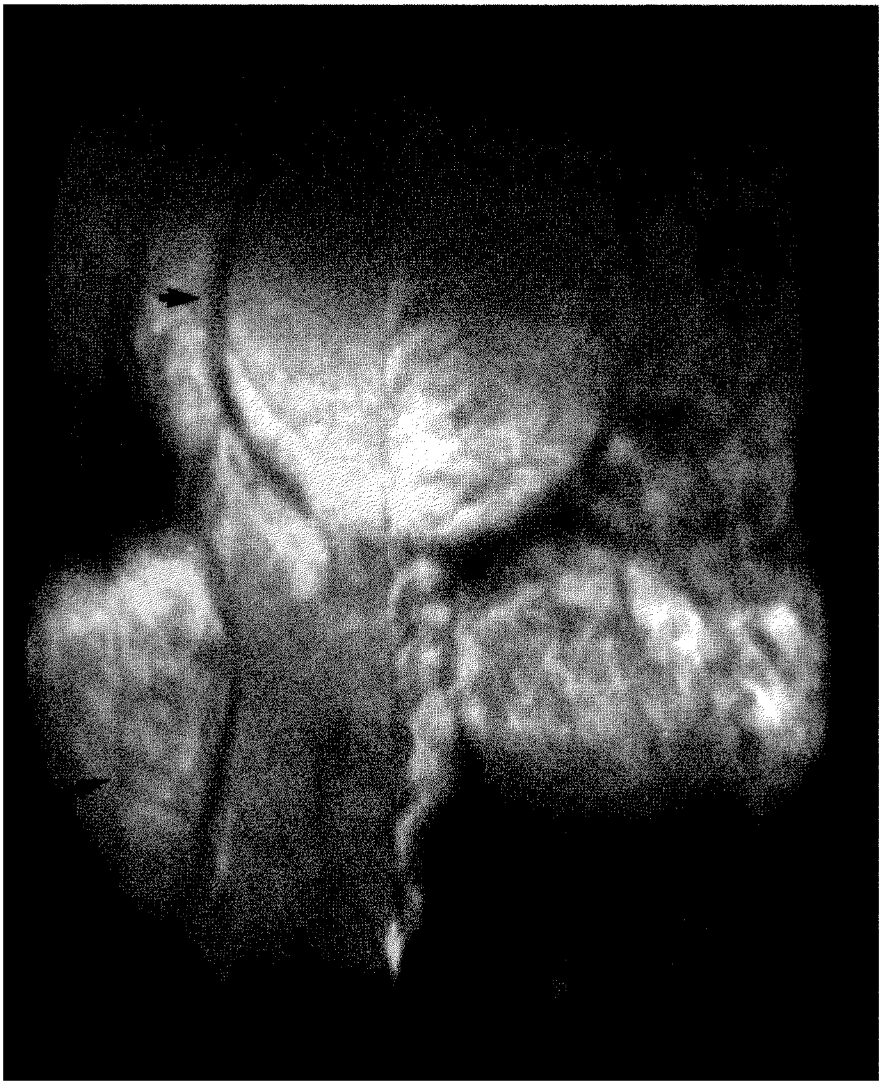

FIGURE 1 Coronal section of MRI showing large mass in white (long arrow) which occupies most of the abdominal cavity. Short arrow represents separate tumor mass in caudate lobe.

oozing from the raw surface of the liver. It was quickly determined that this represented coagulopathic bleeding instead of surgical bleeding and the decision was made to pack the abdomen with abdominal pads and return to the Intensive Care Unit. The patient required two further reoperations over the next four days for removal of packs and further hemostasis.

She recovered and was discharged home after 21 days in the hospital. She remains well in follow up with resolution of her neutropenia and coagulopathy and reports a significant improvement in her quality of life.

### DISCUSSION

Hemangiomas are the most common benign tumors of the liver. In autopsy series the incidence ranges from 0.6 to 7%. Most

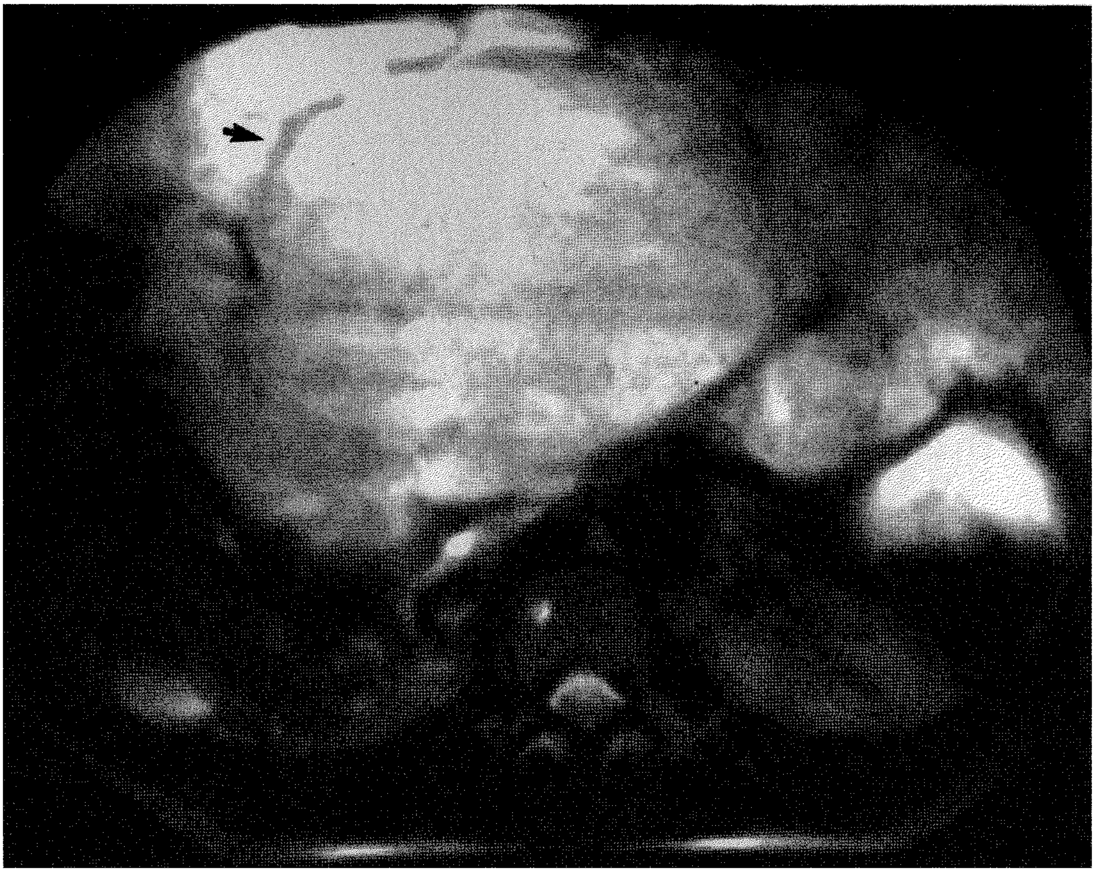

FIGURE 2 Cross section of MRI demonstrating mass in white which occupies entire left lobe of liver and segments V and VIII of right lobe. Short arrow shows caudate mass. Long arrows demonstrates right hepatic vein.

hemangiomas are small, solitary and are seldom symptomatic. Giant hemangiomas are defined as those larger than 4 cm and occur most frequently in women in the fourth and fifth decades and can reach enormous proportions [4]. These may present with symptoms varying from slight discomfort in the upper abdomen to spontaneous rupture of the hemangioma. More rarely, as in the current case, hemangiomas present with consumption coagulopathy [5].

The primary pathophysiologic event of the Kasabach – Merritt syndrome is platelet trapping within the vascular lesion. It may be acute and massive or chronic and low grade. If a compensatory increase in bone marrow megakaryocyte production does not occur, then thrombocytopenia develops. $^{131}$I-labeled fibrinogen studies also demonstrate accumulation of this hemostatic factor in lesions [6].

Non surgical treatment of cavernous hemangiomas of the liver include radiation therapy and embolization and have had mixed success. Reports utilizing radiation therapy have described a partial or complete regression of symptoms in the majority of patients, albeit with rather sketchy details and only clinical assessment of response in the early cases [7]. Data supporting objective tumor regression with radiation therapy are lacking. The optimal dose of radiation is not firmly established but it should be the minimal dose which will alleviate

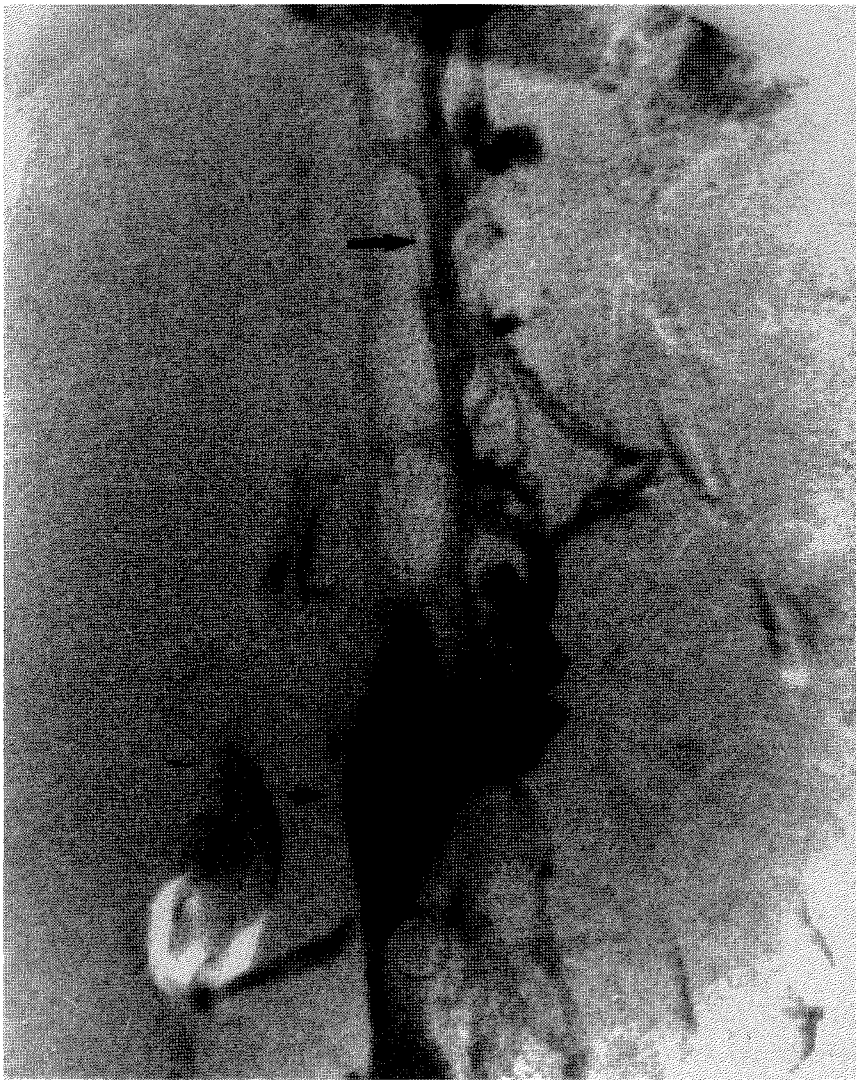

FIGURE 3 Venogram of inferior vena cava is shown. Short arrow represents vena cava below tumor mass. Long arrow represents compression of vena cava with small patent lumen. Note presence of venous collaterals due to chronic caval compression.

symptoms, given that this is the major goal of therapy. Most investigators report utilizing 20–30Gy (in 3–4 weeks) [7]. Given the relative radioresistance of normal vascular endothelium, the mechanism of radiation effect on this tumor is unknown. There is no evidence that radiation therapy relieves the coagulopathy present in patient with giant hepatic hemangiomas and Kassabach–Merritt syndrome. In the case presented here there was no improvement in any symptoms with low dose radiation treatment.

The role of arterial emoblization as a treatment for primary and secondary hepatic neoplasms is well established [8]. For hepatic hemangiomas and Kasabach–Merritt syndrome there are limited data, but one report utilizing intravenous cryoprecipitate plus infusion of intra-arterial thrombin and aminocaproic acid resulted in

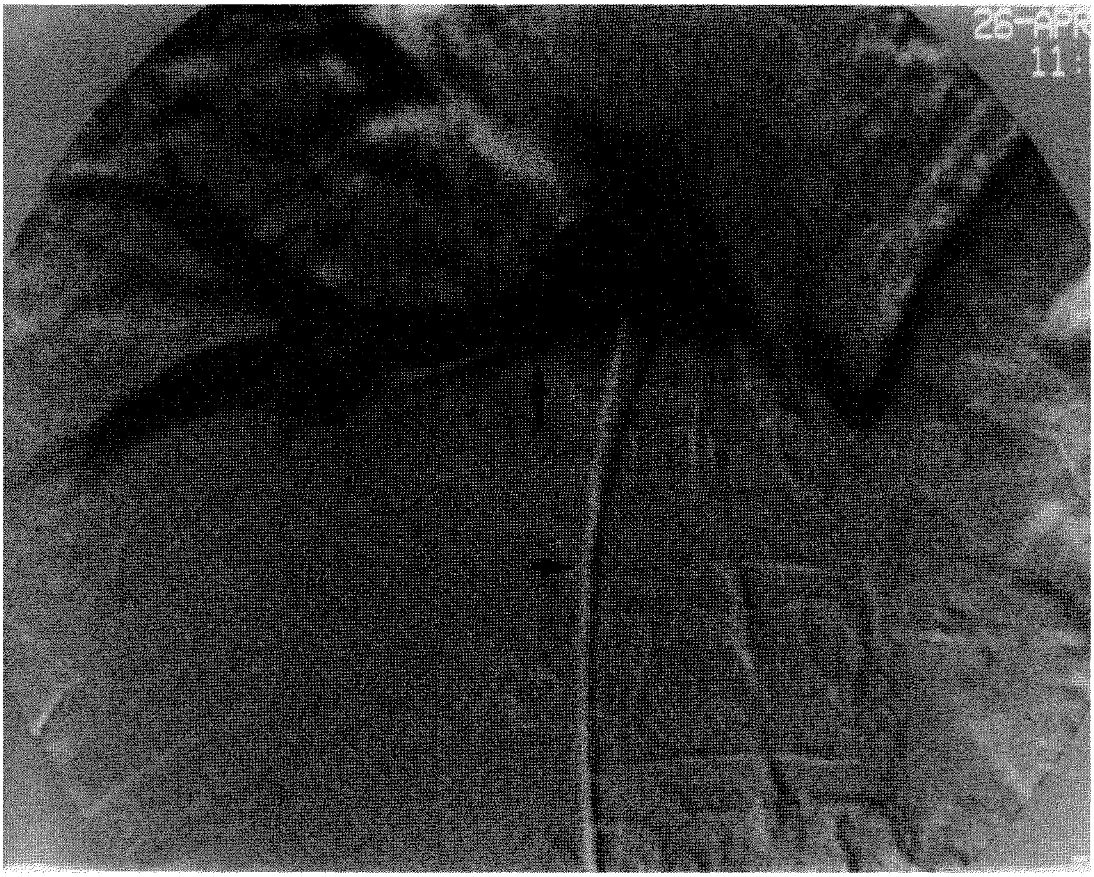

FIGURE 4 Hepatic venogram is demonstrated. Short arrow represents catheter entering hepatic veins. Long arrow represents right hepatic vein which is patent. The left and middle hepatic veins were not visualized due to compression.

complete stasis within the hepatic hemangioma [9]. Unfortunately, in most cases, embolization is only temporarily effective in reducing the size of the hemangioma and improving the bleeding diathesis. As recanalization occurs, there is a concomitant recurrence of the coagulopathy [9, 10]. Thus, the drawbacks to embolization are recanalization and the possibility of a significant reduction in blood supply to an organ, resulting in ischemia and possibly infarction or necrosis.

While surgery appears to be the most effective treatment, whether giant cavernous hemangioma of the liver should be treated conservatively or removed is controversial. Even for symptomatic giant hemangiomas, some authors have advocated long-term follow up. One of the reasons given for this policy is that the morbidity and mortality rates associated with major liver resection have been suggested by some as prohibitive. The almost negligible mortality rate recently reported for elective resection in specialized centers does not support this view [4, 11–13]. Perioperative deaths generally occur during the rare case of emergent treatment of spontaneous bleeding or rupture [14]. Certainly, in those patients presenting with hematologic decompensation, resection should be performed.

Case reports have described orthotopic liver transplantation for giant hemangioma of the liver and Kasabach–Merritt syndrome. In these two reports, hepatectomy was described as a difficult procedure because of the bulk and weight of the tumor. Blood loss was considerable in these reports with transfusion of 14 units of blood in one case and 17 liters of blood products in the other [2,3]. In one of the two cases, a reoperation was necessary due to hemorrhage. While operative mortality for liver transplantation has decreased considerably since its inception, operative mortality is still 10% and 5-year survival approximately 80% for benign disease [15]. In addition, patients must take lifelong medication with frequent follow up.

If surgical treatment is considered, we feel that surgical enucleation rather than liver transplantation is the procedure of choice for cavernous hepatic hemangiomas including those presenting with the Kasabach–Merritt syndrome. This is based on the assumption that coagulopathy and tumor size make it a technically difficult procedure whether enucleation or liver transplantation are performed. In specialized centers, enucleation should be associated with a mortality no higher than that for liver transplantation without the need for lifelong medication and follow up. The extensive blood supply of these lesions requires a meticulous and standardized approach. This has been described elsewhere and depends on prior control of the major extrahepatic feeding vessels, and dissection within the relatively less well perfused rim of compressed tissue through which the feeding vessels supply the lesion [4]. In one report, median blood loss with enucleation for 11 large hemangiomas was 800 ml. Even when the size of the tumor means that most of the right or left lobe has been removed, the disturbance of liver function is insignificant. The technique of enucleation allows safe removal of even very large lesions in most cases.

### *Acknowledgement*

We would like to thank Maria Reyes for her expert assistance.

### *References*

[1] Kasabach, H. H. and Merritt, K. K. (1940). Capillary hemangioma with extensive purpura. *American Journal of Diseases in Children*, **59**, 1063–1070.

[2] Klompmaker, I. J., Slooff, M. J. H., Van Der Meer, J., De Jong, G. M. T., Bruijn, K. M. and Bams, J. L. (1988). Orthotopic liver transplantation in a patient with a giant cavernous hemangioma of the liver and Kasabach–Merritt Syndrome. *Transplantation*, **48**, 149–151.

[3] Longeville, J. H., Hall, P., Dolan, P., Holt, A. W., Lillie, P. E., Williams, J. A. R. and Padbury, R. T. A. (1997). Treatment of a giant haemangioma of the liver with Kasabach–Merritt Syndrome by orthotopic liver transplant: a case report. *HPB Surgery*, **10**, 159–162.

[4] Baer, H. U., Dennison, A. R., Mouton, W., Stain, S. C., Zimmermann, A. and Blumgart, L. H. (1992). Enucleation of giant hemangiomas of the liver. *Annals of Surgery*, **216**, 673–676.

[5] Shimizu, M., Miura, J., Itoh, H. and Saitoh, Y. (1990). Hepatic giant cavernous hemangioma with microangiopathic hemolytic anemia and consumption coagulopathy. *American Journal of Gastroenterology*, **85**, 1411–1413.

[6] Straub, P. W., Kessler, S., Schreiber, A. and Frick, P. G. (1972). Chronic intravascular coagulation in Kasabach–Merritt syndrome. *Archives of Internal Medicine*, **129**, 475–479.

[7] McKay, M. J., Carr, P. J. and Langlands, A. O. (1989). Treatment of hepatic cavernous haemangioma with radiation therapy: case report and literature review. *Australian and New Zealand Journal of Surgery*, **59**, 965–968.

[8] Chuang, V. P. and Wallace, S. (1981). Hepatic arterial embolization in the treatment of hepatic neoplasms. *Radiology*, **140**, 54–58.

[9] Stahl, R. L., Henderson, J. M., Hooks, M. A., Martin, L. G. and Duncan, A. (1991). Therapy of the Kasabach–Merritt syndrome with cryoprecipitate plus intra-arterial thrombin and aminocaproic acid. *American Journal of Hematology*, **36**, 272–274.

[10] Larsen, E. and Zinkham, W. (1987). Kasabach–Merritt syndrome: therapeutic considerations. *Pediatrics*, **79**, 971–980.

[11] Brouwers, M. A. M., Peeters, P. M. J. G., De Jong, K. P., Haagsma, E. B., Klompmaker, I. J., Bijeveld, C. M. A., Zwaveling, J. H. and Slooff, M. J. H. (1997). Surgical treatment of giant haemangioma of the liver. *British Journal of Surgery*, **84**, 314–316.

[12] Weimann, A., Ringe, B., Klempnauer, J., Lamesch, P., Gratz, K. F., Prokop, M., Maschek, H., Tusch, G. and Pichlmayr, R. (1997). Benign liver tumors: differential diagnosis and indications for surgery. *World Journal of Surgery*, **21**, 983–991.

[13] Schwartz, S. I. (1987). Cavernous hemangioma of the liver: a single institution report of 16 resections. *Annals of Surgery*, **205**, 456–465.

[14] Trastek, V. F., Van Heerden, J. A., Sheedy, P. F. and Adson, M. A. (1989). Cavernous hemangiomas of the liver: resect or observe. *American Journal of Surgery*, **145**, 49–53.

[15] Wood, R. P., Ozaki, C. F., Katz, S. M., Monsour, H. P., Dyer, C. H. and Johnston, T. D. (1994). Liver transplantation, the last 10 years. *Surgical Clinics of North America*, **74**, 1133–1154.

